# A Perspective of Coagulation Dysfunction in Multiple Sclerosis and in Experimental Allergic Encephalomyelitis

**DOI:** 10.3389/fneur.2018.01175

**Published:** 2019-01-14

**Authors:** Domenico Plantone, Matilde Inglese, Marco Salvetti, Tatiana Koudriavtseva

**Affiliations:** ^1^S.O.C. Neurologia, Ospedale San Biagio, Domodossola, Italy; ^2^Department of Neurology, Radiology and Neuroscience, Icahn School of Medicine at Mount Sinai, New York, NY, United States; ^3^Department of Neuroscience Mental Health and Sensory Organs (NEMOS), Sapienza University, Sant'Andrea Hospital, Rome, Italy; ^4^IRCCS Istituto Neurologico Mediterraneo (INM) Neuromed, Pozzilli, Italy; ^5^Department of Clinical Experimental Oncology, IRCCS Regina Elena National Cancer Institute, Rome, Italy

**Keywords:** coagulation, neuroinflammation, multiple sclerosis, neuromyelitis optica spectrum disorders, thrombosis

## Abstract

A key role of both coagulation and vascular thrombosis has been reported since the first descriptions of multiple sclerosis (MS). Subsequently, the observation of a close concordance between perivascular fibrin(ogen) deposition and the occurrence of clinical signs in experimental allergic encephalomyelitis (EAE), an animal model of MS, led to numerous investigations focused on the role of thrombin and fibrin(ogen). Indeed, the activation of microglia, resident innate immune cells, occurs early after fibrinogen leakage in the pre-demyelinating lesion stage of EAE and MS. Thrombin has both neuroprotective and pro-apoptotic effects according to its concentration. After exposure to high concentrations of thrombin, astrocytes become reactive and lose their neuroprotective and supportive functions, microglia proliferate, and produce reactive oxygen species, IL-1β, and TNFα. Heparin inhibits the thrombin generation and suppresses EAE. Platelets play an important role too. Indeed, in the acute phase of the disease, they begin the inflammatory response in the central nervous system by producing of IL-1alpha and triggering and amplifying the immune response. Their depletion, on the contrary, ameliorates the course of EAE. Finally, it has been proven that the use of several anticoagulant agents can successfully improve EAE. Altogether, these studies highlight the role of the coagulation pathway in the pathophysiology of MS and suggest possible therapeutic targets that may complement existing treatments.

## Introduction

Multiple sclerosis (MS) is an inflammatory demyelinating and degenerative disease of the central nervous system (CNS) characterized by neuroinflammation and neurodegeneration and affecting prevalently women (1). Most commonly, MS begins with a relapsing–remitting course with alternation of clinical relapses and remissions (2). With time, most of these cases switch to a secondary progressive phase with steady accumulation of disability. In a lower percentage of patients (about 20%) the disease is progressive from the beginning and is defined as primarily progressive form. Experimental autoimmune encephalomyelitis (EAE) is the most studied animal model of MS (3). It is possible to induce EAE in mice by immunization with spinal cord homogenates or by passive transfer of sensitized T cells.

Several recent studies have highlighted the importance of the interplay between the activation of the coagulation cascade and neuroinflammation, suggesting that coagulation factors are crucial not only for the activation of the acute hemostatic cascade, but have a broader role involving neurodegeneration and neuroinflammation (4–20).

We will review this evidence, trying to offer an overview of possible targets that may complement existing immunomodulatory therapeutic tools.

## History

The role of vascular thrombosis due to increased coagulation in MS has been taken into consideration since its first description based on histopathological observations. In 1882, Ribbert postulated that MS lesion could be consequent to central vascular thrombosis due to bloodstream infection (21). This hypothesis was supported by Pierre Marie, who thought that infections have a causative role in MS through the induction of brain vascular damage and thrombosis (21). Thereafter in 1930–40s, Tracy Putnam pointed out venular thrombosis as the primary MS cause based on histologic and experimental observations (22). He observed thrombi in acute lesions with small plaques surrounding the engorged veins. He occasionally observed thrombi also in other body organs of MS patients. Moreover, Putnam found that most MS patients had a peculiar defect of the clotting mechanism, suggesting that thrombosis was not consequent to the vessel wall injury but to blood alterations such as an increase in fibrinogen.

The first studies on serum coagulation factors were conducted in small MS patient cohorts and led to conflicting results. The clotting time was found shortened (23), normal (24) or prolonged (25). There was disagreement also regarding the prothrombin time since it was similarly reported either shortened (26), prolonged, (27, 28) or normal (25, 29). Putnam (30) and Persson (31) showed an increase of fibrinogen especially during the exacerbations while other studies found it to be normal in 9 patients in active clinical progression except one (25) as well as in 33 MS patients except two patients with increased fibrinogen, of which one examined during a relapse (32).

Furthermore, thrombocytes were studied in MS with divergent results. One study reported a short clot retraction time during acute disease exacerbation (33), whereas it was found to be normal or prolonged in other works (25, 32). An elevated platelet adhesiveness and a short clot retraction time was found mainly during acute disease exacerbation (33). Fog et al. found the reduction in thrombocyte count during the disease exacerbation and its increase during the clinical improvement (34) whereas other authors found thrombocyte count normal in MS patients (24, 29, 32) or reduced in several patients in phase of disease remission (32). Persson found an increase in adhesive platelets in the prodromal phase of thrombosis, which rapidly decreased during the thrombi formation (35). Similarly, Wright et al demonstrated increased adhesiveness of platelets in acute and severe cases compared to controls suffering from other neurological disorders and reporting normal values of platelet adhesiveness (36). However, the possibility that steroid therapy could influence this adhesiveness was not excluded. Another explanation for the negative results related to platelet adhesiveness in MS patients found by Field and Caspary could be presence of edetic acid (EDTA) in the test tubes which is known to reduce platelet adhesiveness (37).

Interestingly, Putnam treated his MS patients with Dicumarol in the 1940s and concluded that only patients with acute relapses benefited from this therapy (21). All in all, the enthusiasm for anticoagulant therapy in the scientific community waned in the following years because of its doubtful effectiveness (21).

At the same time, in the 1930s the pathological MS research radically changed its own course after the development of EAE, a prototypic model of MS, through the immunization of susceptible animals with CNS components. Since then, the majority of the studies on disease pathophysiology focused on immunological mechanisms (38). While the results of genome-wide association studies (GWAS) (39) and the success of treatments based on immunological targets reinforce this hypothesis, other studies still suggest a key role for a dysfunction of coagulation, possibly linked to the ongoing inflammation, in CNS autoimmunity (9–11, 15, 40–42).

## Coagulation Cascade Summary

Coagulation is a complex process involving blood changes that lead to the formation of a blood clot (43). It is classically aimed to ensure haemostasis, an important biological process that contrasts bleeding after vessel injury. However, coagulation is activated not only by direct vascular injury but also by functional injury due to hypoxia, sepsis, malignancy, inflammation, etc (44, 45). A pathological imbalance of haemostasis may lead to intravascular thrombosis despite the coagulation process is controlled by several inhibitors limiting the clot formation. However, in certain conditions thrombosis is a physiological process called “immunothrombosis” involving an intrinsic effector mechanism of innate immunity (46). Immunothrombosis is specifically activated by either blood-borne pathogens or circulating altered-self components on a local platform consisting of fibrin, monocytes, neutrophils, and platelets contributing to pathogen recognition. Innate immune cells such as monocytes, neutrophils, dendritic cells participate actively in this process propagating fibrin formation and triggering platelet activation. This process contrasts either tissue invasion, dissemination, or survival of pathogens. The delimitation of immunothrombosis to only a restricted number of microvessels likely ensures a sufficient overall organ perfusion.

Briefly, two traditional coagulation cascade pathways, so-called intrinsic, and extrinsic, lead to the same final common pathway of factor X and thrombin ending with fibrin formation. These coagulation pathways are a series of reactions converting the inactive precursors to active ones in order to catalyze the next reaction in the cascade. Majority of clotting factors are precursors of proteolytic enzymes known as zymogens that circulate in an inactive form.

Platelets exert potent procoagulant functions via the calcium-dependent cell-surface exposure of phospholipids such as phosphatidylserine, which act as cofactors for the proteolytic reactions triggered by coagulation factors. Coagulation process, in turn, fosters platelet activation and accumulation, mainly through the protease thrombin, which promotes platelet activation by both cleavage and activation of platelet's proteinase-activated receptors (PAR). Platelets early aggregate to form a “platelet plug” to close provisionally the vessel wall injury. This platelet adhesion to subendothelial surface is reinforced by von Willebrand factor (vWF), which is a glycoprotein present in blood plasma and produced in endothelium, megakaryocytes, and subendothelial connective tissue. Activated platelets release into the plasma the contents of their granules, which activate other platelets.

In the extrinsic *tissue factor* (TF) pathway, after vessel damage blood-based coagulation factor VII links with TF, which is present in the subendothelial tissue and fibroblasts as well as in a smaller quantity in circulating form on monocytes, to form an activated complex TF-FVIIa. FVII is also activated by FXa, FIXa, FXIIa and thrombin. Under some pathologic circumstances, TF is expressed also by monocytes, neutrophils, endothelial cells, and platelets with increased levels of circulating TF-positive microparticles that amplify the process of coagulation cascade. The activated complex TF-FVIIa activates coagulation factors FIX and FX.

Intrinsic *contact activation* pathway, which mainly activates thrombin, begins with formation of the primary complex on exposed collagen by factor XII, high-molecular-weight kininogen, prekallekerin, and factor XI. Endothelial collagen is exposed only in course of endothelial damage. Factor XII convers in active FXIIa that converts FXI into activated FXIa. FXIa further activates factor IX, which acts with its cofactor FVIII to form tenase complex on a phospholipid surface and to activate factor X to FXa.

In common pathway FXa along with its cofactor FVa, tissue phospholipids, platelet phospholipids and calcium forms the prothrombinase complex, which activates prothrombin to thrombin. Thrombin activates FV and FVIII, releasing the latter from its link with vWF. Thrombin further cleaves circulating fibrinogen to insoluble fibrin and activates factor XIII, which covalently crosslinks fibrin polymers incorporated in the platelet plug. This creates a fibrin network the building block of a hemostatic plug. Thrombin has also pro-inflammatory effects exciting the PAR present on monocytes, lymphocytes, endothelium and dendritic cells. In addition, it is the most important platelet activator activating FVIII and FV and their inhibitor protein C in the presence of thrombomodulin (TM).

## Platelets

Various studies have highlighted the contribution of blood platelets to the inflammatory process that characterizes MS. These cells may be involved also in the pathophysiology of other neurological diseases, such as Alzheimer's disease, Parkinson's disease and Huntington's disease (47). The role of blood platelets during the acute and chronic phase of inflammation is not marginal. These cells release proinflammatory mediators, display molecules on their surface with inflammatory functions and interact with endothelial cells and leukocytes (48).

Platelets release several proinflammatory mediators. Three types of secretory granules have been described in platelets: dense granules, lysosomes, and alpha-granules (48). The latter type is the most abundant. They are produced during megakaryocyte maturation and are considered crucial for platelet functions. Hundreds of soluble factors are stored in these alpha-granules, including prothrombin, tissue factor, high molecular weight kininogen, chemokines (RANTES, CXCL1, CXCL4, CXCL5, CXCL7, CXCL8, CXCL12, CCL2, macrophage inflammatory protein 1-alpha), proangiogenic and antiangiogenic proteins, growth factors, and inhibitory proteases [e.g., plasminogen activator inhibitor, alpha2-antiplasmin, antithrombin III (AT III), protein S, protease nexin-2, plasminogen, and tissue factor pathway inhibitor](48). Dense granules store ATP, GDP, ADP, 5-HT, Ca, Mg and histamine, whereas lysosomal granules contain glycohydrolases, and acid proteases (48). Interestingly, platelets synthesize and secrete matrix metalloproteinases (MMPs) as well as tissue inhibitors of MMPs (TIMPs). The main MMP in platelets is MMP-1, which is important because it activates protease activated receptor 1 (PAR-1), which, in turn, is important for platelet aggregation (48). Through the cyclooxygenase (COX) and platelet activating factor (PAF) pathways, platelets are also able to synthesize lipid mediators, including eicosanoids. PAF can also induce the production of IL-1beta in platelets (48).

Platelets express many molecules on their surface that play a role during the inflammatory response. P-selectin, which translocates from the granules to the surface during the platelet activation, can interact with leukocytes and endothelial cells through the interaction with P-selectin glycoprotein-I (PSGL-1). Platelets express also CD40L on their surface and release its soluble form during activation. The latter, (49) together with PAF (50) and MMPs (51), is crucial in order to increase the permeabilization of the blood brain barrier (BBB).

Whether platelet activation is a primary event of MS pathogenesis or it is secondary to endothelial injury is still matter of debate (52). Platelets interact with leukocytes at the endothelium of the BBB by releasing the adhesion molecule PECAM-1 that triggers leukocyte infiltration (53). Moreover, PAF disrupts endothelial BBB junctions and PAF receptors are up-regulated in MS lesions (53).

Platelets are among the first cell types to begin the inflammatory response in the CNS in the acute phase of MS immune response and may be important to trigger and amplify it by producing significant amounts of IL-1alpha (54).

Finally, activated platelets can produce large amounts of Reactive Oxygen Species (ROS) and Reactive Nitrogen Species (RNS) that can cause damage to proteins, lipids and nucleic acids, leading to death of CNS cells. Importantly, CNS has low antioxidant defenses and the composition of myelin seems particularly vulnerable to ROS (55).

The importance of platelets in MS and in EAE has been demonstrated also in experimental settings. Platelet depletion has been found to ameliorate the course of EAE (56). Moreover, during EAE, platelets have been demonstrated to activate in response to sialated glycolipids integrated into neuronal and astroglial lipid rafts found within the BBB (57).

Starossom et al demonstrated that glatiramer acetate (GA), a disease modifying treatment for MS, significantly inhibited thrombin-induced activation of human and mouse platelets (58). They showed that GA was able to inhibit calcium influx, upregulation of CD62P and other markers of activation and aggregation in human and mouse thrombin-activated platelets. They also found that GA significantly reduced platelet-induced upregulation of CD86 and MHC class II on macrophages, leading to a decreased platelet-mediated activation of macrophages.

## Thrombin

Thrombin is a 36-kDa serine protease, a key enzyme in the coagulation cascade formed after cleavage of its precursor, prothrombin (with a molecular weight of ~72-kDa), by the coagulation factor Xa (59). Prothrombin contains gamma-carboxyglutamic acid, is synthesized in the liver and released into bloodstream. It can be activated by vascular injury, through limited proteolysis following upstream activation of the coagulation cascade. Serum inhibitors and its own action regulate the activity of thrombin. With its procoagulant and anticoagulant functions, the role of thrombin is pivotal in thrombosis and haemostasis. However thrombin has also hormone-like properties that can influence many cells, including platelets, lymphocytes, neurons and astrocytes (60).

Thrombin converts fibrinogen into fibrin and activates factor XIII, affecting the cross-linking of fibrin monomers to produce a stable fibrin clot. Thrombin's function is peculiar as it has both procoagulant and anticoagulant functions. The latter is mediated through binding to TM, a receptor protein expressed on the endothelial membranes, triggering a series of reactions that lead to fibrinolysis. The endothelial protein C receptor (EPCR), shed from the endothelial cells by inflammatory mediators and thrombin, increases protein C activation by the thrombin-TM complex, and inhibits leukocyte extravasation. Interestingly, TNF alpha can down-regulate EPCR, and TM (61).

Depending on the concentrations, thrombin has been demonstrated to have both neuroprotective and pro-apoptotic effects (6, 62). At low to moderate concentrations, thrombin is neuroprotective for hippocampal neurons and astrocytes that lose their star morphology and maintain their supportive role in the production of glutathione and in the reduction of glutamate (7). On the contrary, at high concentrations, thrombin is able to induce cell death (63). In fact, after exposure to high concentrations of thrombin, astrocytes become reactive and lose their neuroprotective and supportive functions, microglia proliferate and produce reactive oxygen species, IL-1β, and TNFα. Moreover, high concentrations of thrombin may produce axonal damage and retraction, intracellular calcium upregulation, and finally cell death. Furthermore, thrombin can induce BBB damage by digestion of extracellular matrix mediated by MMPs (7).

Another function that thrombin has in common with activated factor VII (FVIIa) and FXa is the activation of PARs family proteins, expressed on the surface of several tissues (preferentially PAR1 and PAR3 rather than PAR4 due to their hirudin-like motif) (64) and involved in hemostasis, phlogosis, cancer development, and embryologic differentiation (65).

There are two main thrombin inhibitors: Protease nexin 1 (PN-1) and ATIII. PN-1 is a 47 kDa serine protease inhibitor (SERPIN) that acts as a suicide substrate for thrombin and urokinase-type plasminogen activator (66). It represents the most abundant and potent endogenous brain thrombin inhibitor (67). The expression of PN-1 is high in the brain and this glycoprotein is secreted by glial cells (68) and neurons (69). ATIII is also a SERPIN normally expressed in the liver and at low levels in brain tissue (70, 71). ATIII is a non-vitamin K-dependent protease that inhibits the activity of thrombin and factors IXa and Xa. These SERPINs have been demonstrated to be highly expressed in mice with EAE. The expression of PN-1 in the brain of the mice with EAE peaks at day 8 post-immunization (during the preclinical phase), whereas ATIII peaks at day 13, when the mice experience the highest clinical score and correlate to the disease severity (6).

Significantly higher plasma levels of both prothrombin and factor X have been found in relapsing-remitting MS whereas increased levels of prothrombin have been found in secondary-progressive MS patients compared to healthy controls (9). Conversely, no significant difference was found between controls and patients with both primary-progressive MS and NMOSD. Interestingly, relapse free time negatively correlated with level of either prothrombin, factor XII, or factor X indicating disease exacerbation as a condition characterized by increased coagulation activity (9). Similarly, the speed of thrombin generation was found faster in relapsing-remitting than in primary progressive MS or healthy controls and correlated with time from clinical diagnosis likely reflecting the differential active proinflammatory state in each MS subtype (72). Dermatan sulfate and heparin inhibit the generation of thrombin activity and both have been demonstrated to be effective therapeutic agent for EAE (73–75).

Drugs available to block thrombin action include heparins, hirudins (lepirudin and bivalirudin), vitamin K antagonists and a new generation of direct thrombin inhibitors such as dabigatran and argatroban.

## Fibrinogen

Fibrinogen is a soluble 340-kDa glycoprotein comprised of three distinct polypeptide chains: A-alpha, B-beta, and gamma produced in the liver by the hepatocytes (76). Plasma concentration of fibrinogen is 2–4 g/L, and its half-life is about 4 days (77). Fibrinogen represents an acute-phase reactant, therefore its plasma concentrations increase during inflammatory response. Thrombin cleaves off fibrinopeptides A and B from the fibrinogen molecule, exposing multiple polymerization sites, leading to the polymerization, formation of insoluble and stable fibrin clot and finally, with the involvement of circulating platelets, formation of a platelet plug (78). Platelets bind to the C terminus of fibrinogen's gamma-chain binds, through their surface αIIbβ3 integrin receptor, facilitating the formation of a platelet plug (41).

The deposition of fibrin is frequently associated with inflammation (40) and fibrin can increase the expression of several cytokines which, in turn, modulate cell adhesion, and migration (79). The pattern of fibrin deposition in MS coincides with the areas occupied by demyelinating lesions (80), and with the areas characterized by axonal damage (81). Interestingly, fibrin deposition may precede the formation of demyelinating lesions (82–84).

Plasminogen and fibrinogen were found to be lower in MS compared to healthy controls (85). These results have been explained by a possible fibrinogen consumption and fibrin formation due to activation of coagulation cascade leading to up-regulation of fibrinolytic system with both increased plasmin's and reduced plasminogen's levels. A recent study showed in patients with both clinically isolated syndrome (CIS) and relapsing-remitting MS that a high plasma fibrinogen levels had a high specificity and specificity, but a low sensitivity for detection of active lesions on MRI during relapses supporting a role of fibrinogen on the development of MS lesions (86). A microarray study has demonstrated the presence of fibrinogen transcripts in chronic lesions of MS patients (87). Fibrinogen is able to directly activate microglia *in vitro* and increase its phagocytic ability (88). The importance of fibrinogen, especially in the early phases of MS, has been postulated and eventually demonstrated in mice with EAE, in which the leakage of fibrinogen from the BBB is crucial for microglial activation (89). The deposition of fibrin interferes with axonal regeneration (4) and pharmacologic removal of fibrin in EAE mice has been demonstrated to suppress disease development and improve the resulting disability (90, 91). Fibrinogen can bind to members of three major families of integrins, beta-1 (alpha5-beta1), beta-2 (CD11b/CD18 and CD11c/CD18), and beta-3 (alpha-v-beta3), that are expressed by leukocytes on their surface (92).

The conversion of fibrinogen to insoluble fibrin exposes the cryptic epitope γ377–395. This epitope in crucial for the binding of fibrin to the integrin receptor CD11b/CD18, expressed by microglia (41). Fibrinogen induces release of ROS in microglia and its signaling through CD11b/CD18 is necessary for the formation of perivascular microglial clusters and axonal damage in EAE (89). By the activation of CD11b/CD18 pathway, fibrinogen can stimulate the production of tissue factor (93) and Tumor Necrosis Factor (TNF) (94) by monocytes. Furthermore, the binding of fibrinogen to CD11b/CD18 can result in activation of extracellular signal-regulated kinase 1/2 (ERK1/2) or the phosphoinositide-3 kinase (PI3K) pathway, important for neutrophil survival (95). Nuclear factor kappa B (NF-kappaB) pathway is also activated by fibrinogen and results in increased production of IL-1alpha in monocytes (96).

Mice with EAE, treated with pharmacological depletion of fibrinogen showed a direct reduction of microglia activation (88, 97). An interesting transgenic mouse model (Fib-gamma^390−396A^), characterized by the suppression of the interaction of fibrinogen with CD11b/CD18 was studied in order to analyse the exact role of fibrinogen in EAE. The Fib-gamma^390−396A^ mice with EAE had better clinical scores, decreased inflammation, increased survival rate, and improved motor function than laboratory controls.

A new generation of inhibitors of the coagulation pathway, for example inhibiting fibrinogen binding to CD11b/CD18, with decreased haemorrhagic side effects have been proposed as future treatments of chronic inflammatory diseases, including MS (15, 41). Recently, fibrin-targeting immunotherapy with monoclonal antibody 5B8, targeted against the cryptic fibrin epitope γ_377−395_, has been demonstrated to inhibit autoimmunity- and amyloid-driven neurotoxicity without globally suppressing innate immunity or interfering with coagulation in MS and Alzheimer's disease (42).

## Fibrinolytic System And Anticoagulant Pathways

Several studies have documented presence of products of the fibrinolytic system in MS. Plasminogen is a 93-kDa single chain glycoprotein with an average plasma concentration of 0.2 mg/mL (98). Tissue plasminogen activator (tPA) is a 69-kDa glycoprotein consisting of 527 or 530 amino acids, released as a single chain enzyme, with an average plasma concentration of 5–10 ng/mL (98, 99). Urokinase plasminogen activator (uPA) exists in two forms with different molecular weight: high molecular weight uPA [54 kD], and low molecular weight uPA [33 kD], with a plasma concentration of 1 ng/mL (98). The binding of uPA to its receptor (uPAR) is crucial for the activation of plasminogen to plasmin (100). Leukocytes constitutively express uPAR and the presence of soluble forms of uPAR has been associated with BBB disruption in neurological diseases (101).

Both tPA and uPA convert plasminogen to plasmin and, through a positive feedback mechanism, plasmin cleaves both tPA and uPA, transforming them from their single chain forms to the more active double-chain forms (102). Fibrin represents the major plasmin substrate and enhances plasmin generation by binding both plasminogen and tPA on its surface, increasing also the affinity between tPA and plasminogen (102). Plasmin cleaves fibrin, generating soluble degradation products.

Samples of CSF from MS patients have increased tPA activity as compared to control subjects and the increase in tPA activity correlates with the disease progression (103, 104). In mice with EAE, tPA is detected in macrophages of inflammatory cuffs in the spinal cord (105), and tPA mRNA and protein expression are upregulated, also in neurons (106, 107). Moreover, it has been shown that in MS and in EAE, the uPA, and tPA mediated activation of ubiquitous plasminogen represents a key step in the activation cascade of the four classes of matrix MMPs: collagenases, stromelysins, membrane-type MMPs, and gelatinases. MMPs contribute to the extravasation of circulating lymphocytes and monocytes, by modifying matrix components and can generate encephalitogenic peptides from myelin basic protein (107).

Neurons and microglia in the CNS express tPA (108) and it has been shown that tPA has also an interesting function in neural plasticity. In fact, tPA system has a significant role also in brain tissue remodeling and cell migration (109, 110). tPA is secreted during axonal growth and regeneration, facilitating nerve outgrowth through a tissue matrix (111). tPA levels are reduced in mature brain, with highest levels found in the dentate gyrus and cerebellum (112). tPA mRNA expression is enhanced during cerebellar motor learning tasks in rats and this is considered as a mechanism of synaptic plasticity (113).

In contrast with the increased tPA activity in the CSF from MS patients, tPA deficient mice experienced an early and a more severe and acute EAE as compared to wild-type controls. On the contrary, uPAR deficient mice experienced a delayed and less acute EAE, with a delayed but steadily increased infiltration of inflammatory cells (114). These data highlight the complex role of tPA and uPAR in the pathogenesis of EAE by regulating fibrin deposition at sites of inflammation and cell trafficking into the CNS (114).

Protein C is a vitamin K-dependent zymogen of a serine protease. Protein C is activated by thrombin when both bind to endothelial cell TM. The endothelial protein C receptor (EPCR) also binds protein C. Activated protein C (APC) is a natural anticoagulant. With its cell membrane localizing cofactor, protein S (PS), APC binds to endothelium and activated platelet membranes and intervenes in degradation of procoagulant factor Va and VIIIa, consequently limiting further thrombin formation (115). Impaired TM-dependent aPC generation aggravates EAE disturbing myelination and mitochondrial function and increasing mitochondrial ROS (116). Soluble TM ameliorates EAE and dampened demyelination in the cuprizone-diet model. Recombinant TM ameliorated the clinical and pathological severity of EAE by suppressing plasma levels of inflammatory cytokines (117). Protein C deficiency can be inherited or acquired and causes an important predisposition to thrombosis. However, the roles of APC are not limited to coagulation. APC helps maintain endothelial cell integrity (118), inhibits leukocyte adhesion and BBB crossing (119), reduces the production of pro-inflammatory cytokines (118, 120–123) and has anti-oxidant properties (124). EPCR has structural similarities with the MHC1/CD1 family of molecules, suggesting further possible roles of the protein C pathway in regulating the immune response (118, 125).

Protein C activity was found reduced in MS patients independently from their lupus-anticoagulant activity or factor Va resistance (126). The role of APC in MS has become matter of debate (125).

## Antiphospholipid Antibodies

In common clinical practice serum reactivity for antiphospholipid antibodies (APLs), reduction of prothrombin time or increase of both fibrinogen and D-dimer (a product of fibrin degradation) are accepted indicators of increased coagulation activity, which may indicate intravascular thrombosis. APLs have been widely studied in MS with conflicting results, in part depending on the type of antibodies used in the assays (127). Recently, most authors agree on a higher APL reactivity in MS than in healthy controls even if it is variable according to different disease forms and phases (127–130). APL positivity in MS patients is associated with a more severe clinical and MRI disease progression supporting the concept that the degree of involvement of coagulation in inflammatory-demyelinating diseases is proportional to disease severity (127, 131). Increased APL reactivity has been found in both relapsing-remitting and secondary-progressive MS compared to healthy controls with the highest APL positive rate (> 50%) during the clinical exacerbations and with its decrease a few months after relapse (132). Interestingly, among a broad different APLs only anti-prothrombin and anti-β2 glycoprotein-I antibodies were independently higher in relapse compared to both remission and secondary progressive phase (132). Furthermore, as an example of the close correlation between neurodegenerative and thrombogenic mechanisms in MS, it was showed that high total and LDL levels of cholesterol in MS patients were significantly associated with both disease duration and disability as well as with anti-annexin V positivity (133). Since hydroxychloroquine, a drug with anti-infective, anti-inflammatory and anti-thrombotic properties, protects the annexin V anticoagulant shield from disruption by antiphospholipid antibodies on phospholipid bilayers (134), annexin V has been proposed as a new attractive therapeutic target in MS (135). Ongoing clinical trials are currently testing the effect of hydroxychloroquine in slowing down the progression of clinical disability in MS (ClinicalTrials.gov identifiers NCT02913157 and NCT03109288).

## Alteration of the Coagulation Pathway in Neuromyelitis Optica Spectrum Disorders

Neuromyelitis optica spectrum disorders (NMOSD) represent a more severe CNS inflammatory-demyelinating disorder than MS, characterized by optic neuritis, longitudinally extensive myelitis, and water channel aquaporin-4 autoantibody positivity (136). There are only a few studies comparing coagulation markers including APLs and thrombotic events between MS and NMOSD. A higher anticardiolipin positive rate was found in NMOSD compared to MS patients, associated with a greater ATIII activity and D-dimer level (137). Farber and co-authors reported a significantly higher association of venous thromboembolism with NMOSD than with MS, within 6 weeks of acute relapse, after its correction for influencing factors such as age, length of stay and ambulatory disability (138). These findings are not surprising since a coagulation activation is greater in so far as there is a more severe disease. Partially common pathogenetic mechanisms mediated by coagulation factors and complement, which are part of innate immunity and activate the adaptive immunity, have been supposed for both inflammatory-demyelinating and thrombotic (e.g., antiphospholipid syndrome) CNS diseases (139).

In a recent study investigating the coagulation status of NMO and MS patients, Zhang and colleagues demonstrated that fibrinogen levels were significantly higher in NMO and MS patients compared to non-inflammatory neurological disease subjects as a control group and that there was no difference between MS and NMO. Moreover, fibrinogen levels were significantly associated with the severity of the disease (19). In another study, Göbel and colleagues showed that fibrinogen level was significantly lower in NMOSD compared to both relapsing-remitting and secondary-progressive MS, albeit NMOSD patient's number was low (10). Undoubtedly, peripheral blood measurement of coagulation factors in organ-specific diseases such as MS and NMOSD has some limitations, however their role in the pathogenesis of these disease is matter of intense debate.

## MS Pathogenic Hypothesis Involving Coagulation Pathways

Although there is only a few histopathological examinations of MS samples from the acute phase of the disease (140–143), they have shown early microglia activation (pre-demyelinating lesion stage) after fibrinogen leakage through the damaged BBB (143), as well as the presence of some clotting factors in chronic active lesions identified by a proteomic approach (144).

Moreover, quantitative contrast-enhanced MRI studies found a low grade of BBB leakage in visibly non-enhancing MS lesions, distinct from a significantly greater BBB damage in visibly enhancing lesions (145). The authors showed that this low grade BBB leakage was not influenced by ongoing immunomodulatory therapies supposing a permanent structural changes of vessel walls in chronic long-standing lesions. The abnormalities in “tight” junctions (TJ) between adjacent endothelial cells, which are part of BBB, were found even in normal-appearing white matter (NAWM) (146). The TJ abnormality was not confined to microvasculature but involved the full range of vessels either in MS lesions or in NAWM by a possible effect of pro-inflammatory soluble mediators such as cytokines acting “a distance” (147). The association of fibrinogen leakage with astrocyte's processes as well as with TJ abnormality was most pronounced in active lesions. Also *ex vivo* pathological-imaging correlations using magnetization-transfer ratio and diffusion-tensor imaging showed subtle abnormalities in NAWM, closes to MS lesions and correlated with diffuse microglia activation along with impaired axonal and myelin integrity (148). Furthermore, dynamic-susceptibility enhanced T2^*^-weighted MRI demonstrated prolonged brain blood mean transit time and decreased cerebral blood flow in both white and gray normal-appearing matter of relapsing-remitting MS patients as well as in NAWM of patients with CIS suggesting a continuum of tissue perfusion slowdown starting from white matter and spreading to gray matter (149).

Similarly, histological studies in chronic MS have showed a small deposition of extravascular fibrin in chronic, non-active MS lesions suggesting a persistent BBB damage (82). This steady BBB dysfunction, likely due to its permanent reparative thickening, could determine a continuous low outflow of soluble mediators and inflammatory cells from blood to CNS. Inflammation in progressive MS occurs in the form of compartmentalized immune reaction behind a closed/repaired BBB leading to a formation of lymph-follicle like structures in the meninges and perivascular spaces (150). These local structures produce cytokines, chemokines, and intrathecal immunoglobulins leading to brain damage and disease progression. Compartmentalized inflammation could in part explain the incongruity between greater brain atrophy and fewer radiological inflammatory lesions in progressive MS.

Based on these observations, we could speculate that “soluble” clotting factors and pro-inflammatory mediators released from platelet's granules may in part mediated MS pathogenesis by innate immune activation and consequent adaptive immune stimulation. They pass persistently and subtly in the CNS due to a long-lasting BBB dysfunction in the course of the disease, and more strikingly during MS relapses through acute BBB damage. The big question remains: what triggers these processes and why they occur only in a subgroup of people?

In the health, CNS “immune privilege” status is determined by BBB integrity together with neurons, glia, and the extracellular matrix, which form the neurovascular unit regulating immune responses in the CNS (151). Cell-contact signals expressed by neurons and glia (as a result of neuronal cell adhesion molecules) inhibit both microglia activation and maturation of antigen-presenting cells. Additionally, neurons produce chemokines, neuropeptides, neurotransmitters, and neurotrophins acting as neuroimmunoregulatory mediators to inhibit microglia activation and limit the survival of activated lymphocytes. The impairment of these cell-contact signals due to neuronal damage depletes CNS homeostatic protective environment increasing neuroinflammation (151). This occurs physiologically in aging due to neuronal loss, genetic mutations, oxidative, or metabolic stress with endoplasmic reticulum and mitochondrial dysfunction.

Neurodegeneration seems to be closely associated with neuroinflammation not only in MS, but also in other neurodegenerative disorders due to a chronic activation of the local innate immunity, and in particular of microglia. Microglia is involved in defense against CNS infections and in cleaning of cell debris and damaged proteins, however, its excessive or prolonged activation may cause tissue damage (151). Furthermore, local innate immune activation largely determines adaptive immune response. In fact, neuroinflammation manifests not only with activation of local microglia, astrocytes, oligodendrocytes but also with a recruitment of peripheral innate immune cells such as natural killer, natural killer T cells, mast cells, granulocytes and γδ-T cells as well as of circulating lymphocytes and myeloid cells from the periphery. Activated microglia, by secreting Il-1α, TNF and C1q, induce reactive A1 astrocytes that induce the death of neurons and oligodendrocytes due to their lost ability to promote neuronal survival, outgrowth, synaptogenesis and phagocytosis in neurodegenerative disorders (152). Mutually, systemic immune activation influences the local innate immunity. Peripheral ongoing and precedent infections determine the so-called “primed” environment that increases CNS susceptibility to injury. Experimental studies showed that peripheral inflammation is associated with disease exacerbations in experimental models of either MS, stroke or other neurodegenerative diseases (151).

The same mediators and cells that are involved in neuroinflammation and neurodegeneration, have provide for CNS repair, growth and development (151). Astrocytes are the major source in the CNS of nerve growth factor and glial cell line-derived neurotrophic factor, which are secreted also by T cells. Microglia/macrophages release growth factors and cytochines stimulating axonal regeneration and oligodendrocyte-precursors maturation. However, as in other neurodegenerative diseases, in MS spontaneous regeneration or self-repair of damaged CNS tissue is inadequate compared to the extent of neuroinflammation and neurodegeneration, which co-exist in different degree depending on many factors including tissue localization, lesion formation stage, disease phase and immune system age (153). In acute and limited CNS injury, neuroinflammation could circumscribe neurodegeneration and stimulate regeneration. Conversely, chronic neuroinflammation leads to increased neurodegeneration that in turn impairs homeostatic protective environment further amplifying neuroinflammation and weakening regeneration.

Recurrent or chronic infections lead to immunothrombosis, which is activated by blood-borne pathogens and circulating damaged self-components (46), and are presumably among the causes of chronic neuroinflammation. There is a continuous crosstalk between the immune system and blood coagulation components, closely inter-correlated, and essential for an effective immune response to limit pathogen dissemination and support pathogen killing and tissue repair (45). However, over-activation of coagulation may induce thrombotic complication, excessive inflammation, and tissue damage. Infections cause the modification of proteins' structure and function by increased oxidative stress. A progressive trend of oxidation of several serum proteins including coagulation factors from remission to relapse was found in relapsing-remitting MS patients (154). Moreover, a possible role of transient virus-BBB interactions during viral infections triggering focal inflammation, BBB breakdown and demyelination in some cases of MS has been previously supposed (155). The study of gene-environment interactions showed the relevant relationship between MS genotype and Epstein Barr virus, however also other viruses may perturb the human molecular system by common and unique virus strategies (156). A Danish nationwide nested case-control study found that children with MS have more infections in the 3 years preceding MS beginning that is influenced also by their immune response to infections (157). It is known that several micro-organisms play a role in MS relapse and pathogenesis. Additionally, a recent review based on Cochrane library guidelines concluded that some micro-organisms such as Human herpesvirus 6, Chlamydia pneumoniae and Torque teno virus have contributed to making MS a chronic progressive disease, but it does not rule out the role of other pathogens in MS progression (158). Finally, by immunohistochemistry using specific antifungal antibodies, the microfoci of fungal structures in CNS tissue sections, which was also positive for bacteria, were observed in MS patients but not in controls supporting the polymicrobial infections as a possible cause of MS (159).

## Conclusions

In addition to histopathological observations on early microglia and astrocyte activation after fibrinogen leakage through the damaged BBB in MS and EAE, many observational and experimental studies in MS and NMOSD showed their association either with pro-thrombotic risk factors, increased prevalence of thrombotic and vascular diseases or involvement of clotting factors as well as of complement and platelets, other components of coagulation cascade.

Taken all together, there is evidence for a role of coagulation in the pathogenesis of both MS and NMOSD. It will be important to better define the exact links between immune response and coagulation pathways dysregulation. The new challenge ahead will be to understand how this interaction converges on recently described mechanisms of neurodegeneration induced by activated microglia and reactive astrocytes. This approach may lead to improved treatment options (e.g., polytherapies), not only for demyelinating diseases but also for other neurodegenerative conditions.

## Author Contributions

DP, MI, MS, and TK all contributed equally to the literature research and writing.

### Conflict of Interest Statement

The authors declare that the research was conducted in the absence of any commercial or financial relationships that could be construed as a potential conflict of interest.
