# Corrigendum: A Perspective of Coagulation Dysfunction in Multiple Sclerosis and in Experimental Allergic Encephalomyelitis

**DOI:** 10.3389/fneur.2019.00210

**Published:** 2019-03-12

**Authors:** Domenico Plantone, Matilde Inglese, Marco Salvetti, Tatiana Koudriavtseva

**Affiliations:** ^1^S.O.C. Neurologia, Ospedale San Biagio, Domodossola, Italy; ^2^Department of Neurology, Radiology and Neuroscience, Icahn School of Medicine at Mount Sinai, New York, NY, United States; ^3^Department of Neuroscience Mental Health and Sensory Organs (NEMOS), Sapienza University, Sant'Andrea Hospital, Rome, Italy; ^4^IRCCS Istituto Neurologico Mediterraneo (INM) Neuromed, Pozzilli, Italy; ^5^Department of Clinical Experimental Oncology, IRCCS Regina Elena National Cancer Institute, Rome, Italy

**Keywords:** coagulation, neuroinflammation, multiple sclerosis, neuromyelitis optica spectrum disorders, thrombosis

In the published article, there was an error regarding the affiliations for Marco Salvetti. As well as having affiliation “3,” he should also have “IRCCS Istituto Neurologico Mediterraneo (INM) Neuromed, Pozzilli, Italy.”

There was also an omission in the affiliation for Tatiana Koudriavtseva. Instead of “Department of Clinical Experimental Oncology, Regina Elena National Cancer Institute, Rome, Italy”, it should be “Department of Clinical Experimental Oncology, IRCCS Regina Elena National Cancer Institute, Rome, Italy”.

The authors apologize for this error and state that this does not change the scientific conclusions of the article in any way. The original article has been updated.

